# Assessing the feasibility and effectiveness of household-pooled universal testing to control COVID-19 epidemics

**DOI:** 10.1371/journal.pcbi.1008688

**Published:** 2021-03-09

**Authors:** Pieter J. K. Libin, Lander Willem, Timothy Verstraeten, Andrea Torneri, Joris Vanderlocht, Niel Hens

**Affiliations:** 1 Interuniversity Institute of Biostatistics and statistical Bioinformatics, Data Science Institute, Hasselt University, Hasselt, Belgium; 2 Artificial Intelligence Lab, Department of computer science, Vrije Universiteit Brussel, Brussels, Belgium; 3 KU Leuven – University of Leuven, Department of Microbiology and Immunology, Rega Institute for Medical Research, Clinical and Epidemiological Virology, Leuven, Belgium; 4 Centre for Health Economics Research and Modelling Infectious Diseases, Vaccine and Infectious Disease Institute, University of Antwerp, Antwerp, Belgium; University of Notre Dame, UNITED STATES

## Abstract

Outbreaks of SARS-CoV-2 are threatening the health care systems of several countries around the world. The initial control of SARS-CoV-2 epidemics relied on non-pharmaceutical interventions, such as social distancing, teleworking, mouth masks and contact tracing. However, as pre-symptomatic transmission remains an important driver of the epidemic, contact tracing efforts struggle to fully control SARS-CoV-2 epidemics. Therefore, in this work, we investigate to what extent the use of universal testing, i.e., an approach in which we screen the entire population, can be utilized to mitigate this epidemic. To this end, we rely on PCR test pooling of individuals that belong to the same households, to allow for a universal testing procedure that is feasible with the limited testing capacity. We evaluate two isolation strategies: on the one hand *pool isolation*, where we isolate all individuals that belong to a positive PCR test pool, and on the other hand *individual isolation*, where we determine which of the individuals that belong to the positive PCR pool are positive, through an additional testing step. We evaluate this universal testing approach in the *STRIDE* individual-based epidemiological model in the context of the Belgian COVID-19 epidemic. As the organisation of universal testing will be challenging, we discuss the different aspects related to sample extraction and PCR testing, to demonstrate the feasibility of universal testing when a decentralized testing approach is used. We show through simulation, that weekly universal testing is able to control the epidemic, even when many of the contact reductions are relieved. Finally, our model shows that the use of universal testing in combination with stringent contact reductions could be considered as a strategy to eradicate the virus.

## Author summary

As pre-symptomatic transmission is an important driver of COVID-19 epidemics (i.e., the virus is transmitted before the infected individual is aware of its infection), contact tracing efforts struggle to fully control SARS-CoV-2 epidemics. For this reason, the use of universal testing, where each individual of the community is tested on a regular basis, has been suggested. However, the large amount of PCR tests that is required to facilitate this approach, remains an important impediment. Therefore, we propose a new universal testing procedure that is feasible with the current testing capacity, where we rely on PCR test pooling of individuals that belong to the same households. We evaluate this universal testing procedure in a fine-grained epidemiological model (i.e., an individual-based model) that covers the Belgian population. Through this evaluation, we assess the procedure’s performance to keep the epidemic under control, while allowing for various contact reductions. We assess the robustness of the model, by analysing different levels of community compliance, and we show that weekly universal testing could prove a successful strategy to control SARS-CoV-2 outbreaks.

## 1 Introduction

The SARS-CoV-2 pandemic has caused over 10 million COVID-19 cases and over 0.5 million deaths around the world, since September 2020 [1]. This infection count is presumably an underestimate due to the large proportion of asymptomatic cases [2]. Presently, there are different vaccine and treatment candidates under development and in different phases of clincal trials, including vaccines that are being approved by government agencies [3–5]. Nevertheless, the control of SARS-CoV-2 outbreaks still predominantly relies on non-pharmaceutical interventions. Whereas, at the start of the pandemic invasive measures such as a full societal lock-down were used to avoid an overflow of the intensive care units [6], later on, many countries aimed to control their local SARS-CoV-2 epidemic using a combination of social distancing, teleworking, mouth masks and contact tracing. Yet, while these measures have the potential to reduce the number of detectable infections below 20 cases per 100.000 individuals per day (https://www.ecdc.europa.eu/en/geographical-distribution-2019-ncov-cases), this still leaves regions prone to local outbreaks, that again require more stringent mitigation measures with societal and economical implications. In the fall of 2020, many countries had problems controlling the virus, including Spain [7], Belgium [8], Israel, and the United Kingdom.

The burden of hospitalisation and COVID-19 related mortality seems to be the major motivation to reduce the number of infections. However, keeping the number of infections as low as possible is in the overall population’s interest, considering reports on COVID-19 related morbidities throughout all age groups, including neurological conditions, persistent post-recovery symptoms, cardiac injury and pulmonary fibrosis [9–12]. However, as pre-symptomatic transmission remains an important driver of the epidemic, it comes as no surprise that contact tracing struggles to fully control SARS-CoV-2 epidemics [13–15]. This is further complicated by the fact that contact tracing is sensitive to the reported number of contacts, which depends on the reporting compliance of the traced individual [14, 15]. The use of universal testing (i.e., testing the entire population of a geographical region) has been suggested as a solution to suppress SARS-CoV-2 epidemics [16–18]. Yet, the number of tests necessary to test a country’s entire population in a reasonable time window, remains a serious impediment to this approach.

In this work, we use PCR test pooling, i.e., we combine a number of samples into a pool and test this pool using a single PCR test. As it is logistically most convenient to test household members at the same time, we construct pools out of individuals that belong to the same household. Additionally, testing household members simultaneously agrees with the fact that household members are prone to infect each other [19]. We consider sample pooling for pools of size 16 and 32, for which recently PCR test sensitivity scores were established [20].

This approach facilitates two isolation strategies. Firstly, *pool isolation*, where we isolate all individuals that belong to a positive PCR test pool, regardless of their individual infection status. Secondly, *individual isolation*, where we determine which of the individuals that belong to the positive PCR pool are positive, through an additional testing step. In this isolation strategy, the individuals in the positive PCR pool are kept in isolation until the individual testing results become available, upon which the individuals that test negative are released from isolation. Thus, in the individual isolation strategy, only the individuals that are responsible for the positivity of the pooled sample are isolated. Both isolation strategies have their advantages and disadvantages. On the one hand, when each individual that belongs to a positive pool is isolated (i.e., pool isolation), this means that negative individuals will be isolated as well, which might have implications for the community compliance with respect to isolation. The pool isolation strategy reduces the need for additional tests, rendering it a preferred strategy when the prevalence is high, and the required number of additional tests is unavailable. On the other hand, when prevalence is low, the number of additional required tests is expected to be low as well, and in such an epidemic phase, performing the additional tests necessary for the individual isolation strategy might prove worthwhile, as it could increase isolation compliance.

For both strategies, it is necessary that the pools have a similar number of households, i.e., that the difference in number of households between pools is minimal, to minimize the number of households that are affected when a pool tests positive. To meet this objective, we devise a heuristic allocation algorithm to assign households to a set of pools of equal size.

We evaluate this universal testing approach in an individual-based epidemiological model in the context of the Belgian COVID-19 epidemic [21] and demonstrate that it is possible to test the whole Belgian population (11 million individuals), in a time span of 1 to 4 weeks. For this, we rely on the testing capacity that was available in Belgium in autumn and winter [22, 23], to accommodate the expected indidence of respiratory infections [24]. While we conduct experiments concerning the implementation of universal testing to test the whole Belgian population, we note that the presented framework can also be used to design reactive policies to control local outbreaks (e.g., cities) [25]. In order to assess the robustness of our universal testing approach, we consider different levels of testing and isolation compliance. Furthermore, we consider different false negative rates of the pooled PCR test and the impact of the pool size.

In this work, we show through simulation, that universal testing is able to control the epidemic, even when many of the contact reductions are relieved. Additionally, universal testing implicitly implements surveillance at a high resolution, resulting in a good estimate of the actual incidence and the heterogeneity of this incidence with respect to geography and age. This detailed view on the state of the epidemic will ensure that emergency signals are picked up more rapidly, enabling a swift response that might avoid more invasive control measures.

We acknowledge that the implementation of universal testing is challenging to organise. Therefore we discuss the different aspects related to sampling and PCR testing, to demonstrate the feasibility of universal testing when a decentralized testing approach is used. Finally, our models show that, in the event that a vaccine would not become available in due time, the use of universal testing in combination with stringent contact reductions, could be considered as a strategy to eradicate the virus.

## 2 Methods

### 2.1 Household-to-pool allocation

Consider a set of households H, where each household h∈H is a set that is comprised of individuals, i.e., household members. From H, we have the total population size,

$$\begin{array}{c} N=\sum_{h\in H}\left|h\right| \end{array}$$ (1)

We aim to allocate the households to a sequence of pools with a fixed capacity *k*:

$$\begin{array}{c} \theta ={\left\langle \theta_{i}\right\rangle }_{i=1}^{\left\lceil \frac{N}{k}\right\rceil }, \end{array}$$ (2)

where each pool *θ*_*i*_ is a subset of H, for which

$$\begin{array}{c} \sum_{h\in \theta_{i}}\left|h\right|\leq k \end{array}$$ (3)

As stated earlier, we aim to construct pools with a similar number of households, and thus our objective is to minimize the difference in number of households between pools:

$$\begin{array}{c} \underset{\theta }{\text{arg}\;\text{min}}\left[\text{max}\right|\theta_{i}\left|-\text{min}\right|\theta_{i}\left|\right] \end{array}$$ (4)

To this end, we formulate a greedy heuristic algorithm (Algorithm 1). Note that we formulate a heuristic, as executing an optimal search algorithm is intractable. The reason is that this allocation setting is related to the bin-packing problem, which is NP hard [26].

First, the households are sorted in a descending order according to their size, resulting in a set $H_{s}$. To initialize, we construct an allocation *θ* of empty pools. Then, in each step of the algorithm, we take away the top item of $H_{s}$ and add it to one of the pools that has the least households and members. Note that, to ensure a realistic logistic setting, this algorithm should be run on a set of households that live in close proximity. As detailed in Section 2.3, we run this algorithm on households that belong to the same province (i.e., an administrative region in Belgium).

From the most fundamental perspective, this algorithm always adds a household to one of the pools that has the least number of households, which is a heuristic that will result in an optimal allocation with respect to the objective stipulated in Eq 4. This heuristic enables us to create an optimal allocation for the populations considered in our experiments (Section 3).

**Algorithm 1**: Household to pool allocation algorithm

**Given**: H, *N* and *k*

**Result**: Find a pool allocation *θ*

1 We sort H as $H_{s}$ in a descending order, according to household sizes.

2 We create a sequence of empty pools $\theta ={\langle \theta_{i}\rangle }_{i=0}^{\left\lceil \frac{N}{k}\right\rceil }$.

3 **for** $h\in H_{s}$ **do**

4  *Find the index of the pool to add household h to*:

5  $I_{\text{min}}\leftarrow {\text{arg}\;\text{min}}_{i}\left|\theta_{i}\right|$

6  $I_{\text{min}}^{\prime }\leftarrow {\text{arg}\;\text{min}}_{i\in I_{\text{min}}}\sum_{h\in \theta_{i}}\left|h\right|$

7  *j* ← Uniformly random sample from $I_{\text{min}}^{’}$

8  *Add household* *h* *to pool* *θ*_*j*_:

9  *θ*_*j*_ ← *θ*_*j*_ ∪ *h*

10 **end**

### 2.2 Individual-based model

**Fig 1:**
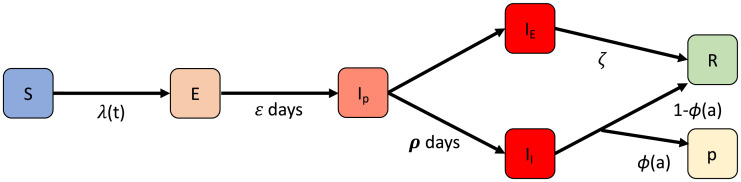
We consider that an individual goes through different phases of infection/disease, which is represented by a SEIR-like state machine. A susceptible individual (S) can become infected, given a time-dependent infection probability λ(*t*). This probability depends on the transmission potential of the virus and the social contact behaviour, which due to contact reduction policies is time-dependent. When infected, the individual becomes exposed (*E*). Once exposed (*E*), an individual goes through an incubation time of *ε* days, after which the individual becomes infectious prior to symptom development (*I*_*p*_). A pre-symptomatic infected individual (*I*_*p*_) will either become asymptomatic (*I*_*a*_), symptomatic with symptoms (*I*_*s*_), after a period of *ρ* days. When asymptomatic (*I*_*a*_) the individual will remain infectious for *ζ* days after which he/she recovers (*R*). When severely symptomatic, the individual will be hospitalized with an age-dependent probability *ϕ*(*a*) or recover without the need for hospitalisation.

We use the *STRIDE* individual-based model to simulate households and the social interactions that take place within and between these households [27]. *STRIDE* has been used to reproduce the Belgian COVID-19 epidemic and to evaluate different strategies to gradually exit the lock-down [21]. This model was able to closely match the data that was observed during the Belgian epidemic (i.e., hospital admissions, serial sero-prevalence) [21, 28, 29]). We present a schematic overview of the infection and disease phases individuals can experience in the model in Fig 1. In this study we use the parametrization shown in Table 1. This parametrization produces a generation interval of 5.16 days, a doubling time of 3 days and a basic reproduction number of 3.41 (confidence intervals in [21]).

**Table 1:** Disease characteristics and references. Transition parameters are discretized to the modelling time step of 1 day. The hospitalization probability is conditional upon being symptomatic. A detailed description on the age dependent proportion of symptomatic cases can be found in the Supplementary section “Age-specific proportion symptomatic cases” of the manuscript by Willem et al. [21].

Parameter	Model value	References
Incubation period	5-7 days (uniform)	[30, 31]
Symptomatic period	4-6 days (uniform)	[31]
Infectious period	5-7 days (uniform)	[31]
Pre-symptomatic infectious period	2-3 days (uniform)	[31]
Hospitalization probability (0-18y)	0.05	[28, 32, 33]
Hospitalization probability (19-59y)	0.03	[28, 32, 33]
Hospitalization probability (60-79y)	0.12	[28, 32, 33]
Hospitalization probability (+80y)	0.60	[28, 32, 33]
Hospitalization delay (0-18y)	3 days	[28, 32, 33]
Hospitalization delay (19-59y)	7 days	[28, 32, 33]
Hospitalization delay (60-79y)	7 days	[28, 32, 33]
Hospitalization delay (+80y)	6 days	[28, 32, 33]
Infectiousness asymptomatic case	50%	[34]
Proportion of symptomatic cases	Age-dependent	[21, 35]
Children’s susceptibility	50%	[21, 36]

We extend the *STRIDE* model to incorporate universal testing as described in Section 2.3. The source code of the *STRIDE* model is available as free (GPLv3) software from GitHub https://github.com/lwillem/stride.

### 2.3 Pooled-households universal testing

We assume to have a fixed set of PCR tests available per day, *T*_*d*_. Each PCR test is used to test one pool of individuals, *θ*_*j*_, as defined in Section 2.1. As we assume that pools have a fixed capacity *k*, this means that we can test up to *k*⋅*T*_*d*_ individuals per day. We define one *testing sweep*, as the period (i.e., number of days) it takes to test the entire population, given the ability to test *k* ⋅ *T*_*d*_ individuals per day. We define universal testing as a repetition of *testing sweeps* to test the entire population.

To plan which households will be tested at which day of a testing sweep, two steps are performed. Firstly, we use the allocation algorithm to appoint households to pools (see Section 2.1). To improve the feasibility with respect to the logistics of the testing procedure, we construct a sequence of pools per province (i.e., an administrative region in Belgium, NUTS-2 level, more information at https://ec.europa.eu/eurostat/web/nuts/background). Secondly, for each of the provinces, we assign the obtained pools to the different days of the sweep, such that on each day of the testing sweep, a portion of the province is tested that is proportional to the province’s population size. This assignment will be used for each of the testing sweeps (i.e., repetitions) that will be conducted.

By testing household members together, our approach facilitates two isolation strategies: *pooled isolation*, i.e., isolating all members of the infected pools, and *individual isolation*, i.e., performing an additional testing step to identify which households are effectively positive. In the case of *pooled isolation*, we isolate each individual for 7 days [37]. In the case of *individual isolation*, we keep the individuals in the pool isolated until it is clear, through individual testing, which individuals are positive. These positive individuals are then kept in isolation until they have been isolated for a total of 7 days [37].

To permit a realistic modelling framework for universal testing, we consider the following parameters: testing compliance *c*_*t*_ (i.e., the fraction of households that will cooperate and allow for the test to be performed), isolation compliance *c*_*i*_ (i.e., the fraction of households that will, when asked to self-isolate, comply to the request), the number of days it takes to get the results from the test *d*_*t*_ and the false negative rate of the PCR test FNR_PCR_. When evaluating the *individual isolation* strategy, we consider the same false negative rate for individual testing, which results in an indivdiual false negative rate that is in line with the work by Firth et al. [38].

Once the test sweep planning has been established, the model will, for each simulated day that universal testing is enabled, iterate over all pools and determine whether a pool tests positive.

For each pool *θ*_*i*_, we first determine which of the households in the pool comply to testing, using a Bernoulli experiment $B\text{ern}(c_{t})$ for each household in *θ*_*i*_. We then determine whether one of the compliant households has an individual that has been infected for at least PCR_delay_ days, such that this infection can be picked up by a PCR test. This is determined based on the infection status of the individuals that make up the household, which is encoded in the state of the individual-based model. We choose a delay of 2 days (i.e., PCR_delay_ = 2), informed by the time onset of the pre-symptomatic infectious period (see Table 1), and in line with earlier work [14, 39]. When positive, we determine with a Bernoulli test $B\text{ern}(1-{\text{FNR}}_{\text{PCR}})$ whether the PCR test will detect it. When detected, we isolate either the infected individuals (individual isolation strategy), or all of the households in pool *θ*_*i*_, with a per-household compliance that is determined by a Bernoulli test $B\text{ern}(c_{i}$).

This procedure thus considers two types of compliance: firstly, we determine whether an individual is compliant to testing, subsequently, when a test is performed and this individual’s pool is found positive, the individual is placed in isolation according to its isolation compliance (with an individually variable isolation compliance). Regarding the universal screening procedure, we thus assume that individuals only go in isolation after being notified of a positive test.

We conduct our experiments by executing 5 stochastic trajectories of the model, for each combination of the parameters we investigate, to assess the stochastic variation of the model.

## 3 Results

We conduct a series of experiments to investigate the proposed universal testing procedure under different assumptions. All of the experiments consider the Belgian COVID-19 epidemic that was fitted to data observed during the epidemic [21]. We assume that individuals are less infectious when asymptomatic (50% reduction in infectiousness) [34]. Furthermore, we assume that 7% of children (i.e., individuals aged 0-19 year) is symptomatic [21]. We use the transmission model from Willem et al., that was calibrated assuming that children are only half as susceptible compared to adults [21]. Given these assumptions, we reproduce the history of the lock-down, to obtain an age-specific immunity profile. To reproduce the lock-down scenario, such that it fits the data that was observed during the Belgian COVID-19 epidemic (i.e., hospitalization and sero-prevelance data) [21], we impose a 75% workplace contact reduction and a 90% leisure contact reduction. Furthermore, we assume that schools (including tertiary education) were closed during lock-down.

To evaluate the household-pooled universal testing procedure, we consider a relieve of the lock-down, at which time we start performing testing sweeps (i.e., repetitions, defined in Section 2.3). We consider this relieve at two time-points: the first of May, with about 50000 active infections, and the first of July, with about 1000 active infections. Note that we consider any infected individual to be an active case, which includes different symptomatic stages (pre-symptomatic, symptomatic, asymptomatic) and individuals that are not aware of their infection status. These two starting points allow us to differentiate between an epidemic with a downward trend that still has a large number of active cases, and an epidemic that is under control, yet prone to experience outbreaks. When the lock-down ends, we assume that the work contact reduction is 50% (compared to 75% during lockdown) [40] and the leisure contact reduction is 70% (compared to 90% during lockdown). Additionally, we open all schools, except for tertiary education. Concerning school openings, we disregard any holidays, to be time-invariant, and thus keep our experiments as generic as possible. Note that all contact reductions, both during and after lockdown are relative to pre-lockdown observations that were obtained in a social contact survey [41].

To explore the efficiency and robustness of our universal testing approach, we consider different values for the universal testing model parameters. Firstly, we consider a pool size *k* ∈ {16, 32}. The pool size impacts the isolation policy, as it dictates the amount of households that need to be isolated when pool isolation is used. We consider the false negative rate of the PCR test, FNR_PCR_ ∈ {0.1, 0.05}. This choice is motivated by the study of Yelin et al. that reports a false negative rate of 10% when using PCR pools up to size 32. Yet, we also consider a lower false negative rate of 5%, regarding the prospect of improved test pool protocols, or the use of smaller pool sizes (e.g., *k* = 16). Secondly, we consider the availability of PCR tests per day *T*_*d*_ ∈ {25k, 50k} (note that k signifies kilo, i.e., 10^3^) as motivated in the Introduction section and further discussed in Section 7. Thirdly, we consider household compliance with respect to testing, *c*_*t*_ ∈ {0.8, 0.9}, and isolation, *i*_*t*_ ∈ {0.8, 0.9}, to evaluate the robustness of our testing framework, when cooperation with the universal testing policy is imperfect. Finally, we assume that the pooled PCR test results can be reported to individuals within 1 day (i.e., *d*_*t*_ = 1). We assume that a one day turn-around time is reasonable, when the extraction of samples and the PCR testing of these samples can be organized in a decentralized fashion. This way, tests can be carefully planned, rather than that they need to be performed on demand (e.g., in the case of contact tracing), and can be collected and analysed in the same location. In Section 5, we discuss these logistic considerations in more detail, to further motivate this reasoning. Furthermore, we conduct a sensitivity analysis considering *d*_*t*_ = {1, 2, 3, 4} in S1 Supplementary information S1.3. We present an overview of the different parameter values in Table 2.

**Table 2:** Overview of the model parameters related to the universal testing framework.

Parameter	Values
Pool size (*k*)	16, 32
PCR false negative rate (FNR_PCR_)	5%, 10%
PCR tests per day (*T*_*d*_)	25k, 50k
Isolation compliance (*c*_*i*_)	80%, 90%
Test compliance (*c*_*t*_)	80%, 90%
Reporting delay (*d*_*t*_)	1 day

**Fig 2:**
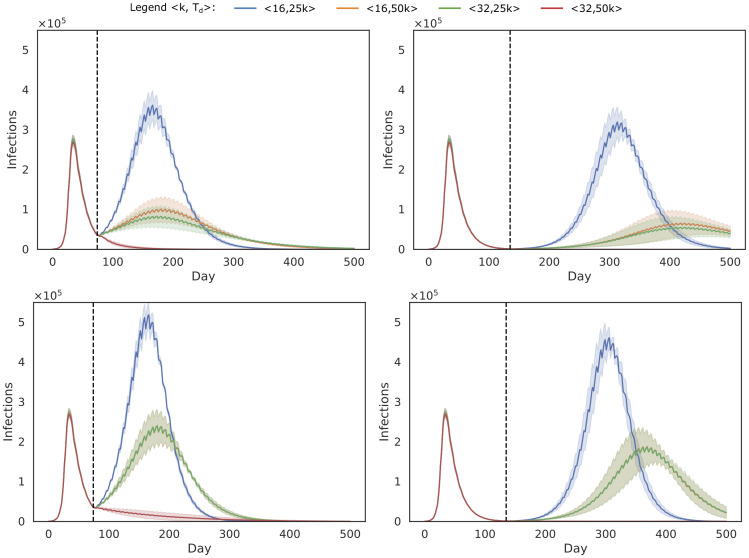
Trends (average and standard deviations) for all combinations of parameters 〈*k*, *T*_*d*_〉, for FNR_PCR_ = 0.1. Universal testing starts at the first of May (left panels) and the first of July (right panels), as indicated by the vertical dotted line, which also marks the end of the lock-down. In the top panels, we follow the pool isolation strategy, where we isolate all individuals that are part of an infected pool. In the bottom panels, we follow the individual isolation strategy, where we identify the infected individuals in positive pool.

To investigate the impact of our household-pooled universal testing approach, we obtain 5 stochastic trajectories of the model, for each combination of these parameters. When analysing these results, we observe that the main trends of the model results are due to the amount of days one sweep takes to complete. As this is determined by the pool size *k* and the number of PCR tests that are available per day *T*_*d*_, we show these trends by aggregating all model results (i.e., test and isolation compliance) per combination of 〈*k*, *T*_*d*_〉, for each of the considered false negative rates FNR_PCR_. In the top panels of Fig 2, we show the trends for both starting points (May and July) and a false negative rate FNR_PCR_ = 0.1 (i.e., the least optimistic of the considered false negative rates), and we follow the pool-isolation strategy, where we isolate all individuals that are part of an infected pool. This figure demonstrates that, in our simulations, we can only achieve proper control of the epidemic, i.e., when *k* = 32 and *T*_*d*_ = 50000. This parameter combination allows for the highest number of individuals to be tested per day (i.e., the whole population in one week), which shows a rapid decrease of the number of infections. Similar trends were observed when the false negative rate is 5%, and these results are shown in S1 Supplementary information S1.1. To demonstrate the performance gradient between weekly testing and bi-weekly testing, we also show results for *k* = 32 and *T*_*d*_ ∈ {25k, 30k, 35k, 40k, 45k, 50k} in S1 Supplementary information S1.5.

We compare these results to the individual isolation strategy in the bottom panels of Fig 2, where we determine which of the individuals that belong to the positive pool are actually positive. Overall, the results with pool isolation and individual isolation are similar. However, due to the additional test that is required for each of the individuals that belong to a positive pool, and the false negative rate that is again associated with it, the performance of individual testing is lower. We observed similar trends for the 5% false negative rate, and these results are shown in S1 Supplementary information S1.1.

**Fig 3:**
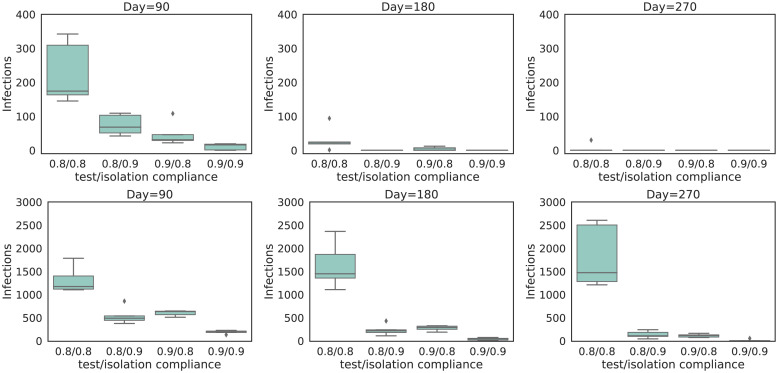
Distribution of the number of infections for the experiments when the lock-down ends on the first of July, in different scenarios of compliance for testing and isolation. We show results for the pool isolation strategy (top panels) and individual based isolation strategy (bottom panels). We show the number of infections at three different time points. i.e., 90 days (left panels), 180 days (middle panels) and 270 days (right panels) after the start of the universal testing procedure. These results consider a weekly universal testing procedure (i.e., *k* = 32 and *T*_*d*_ = 50000) and a FNR_PCR_ = 0.1. Each box represents a combination of test and isolation compliance.

To demonstrate the influence of test and isolation compliance on the decrease in infections, we show box-plots (Fig 3) that depict the number of infections at three different time points (i.e., 90 days, 180 days and 270 days after the start of the universal testing procedure), for the experiments when the lock-down ends on the first of July. In Fig 3, we show the results for the two isolation strategies, respectively pool isolation (top panels) and individual isolation (bottom panels), for the weekly universal testing procedure (i.e., *k* = 32 and *T*_*d*_ = 50000). In Fig 3, we show results for a false negative rate of 10%. Similar trends were observed for a false negative rate of 5% and these results are shown in S1 Supplementary information S1.1. These results demonstrate that we converge to zero cases for both isolation strategies, except when individual isolation is used, and the compliance of both test and isolation is below 80%. Due to the additional test that is required for each of the individuals that belong to a positive pool, and the false negative rate that is again associated with it, individual isolation exhibits a lower convergence rate. Another important consideration here, is the difference in compliance between the two isolation strategies, as we argue that individuals will more likely comply when they know that they are infected. We note that, when compliance is higher, the effect of individual isolation is less pronounced.

Note that, under the leisure and work contact reductions that we assume, the cases converge to zero over a time span that covers many months. In Section 4, we show that the number of cases can drop more quickly, when the contact reduction is more pronounced.

**Fig 4:**
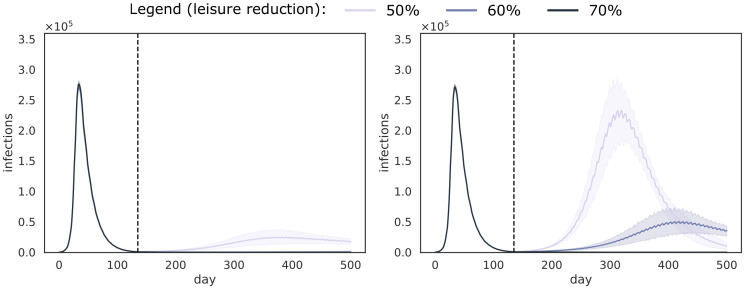
Trends for different leisure contact reductions, when performing weekly universal testing. We assume that universal testing starts (and lock-down ends) on the first of July, as marked by the vertical dotted line, and that FNR_PCR_ = 0.1. We consider both isolation strategies: pool isolation (left panel) and individual isolation (right panel). The curves show a line that depicts the average over the trajectories of the result aggregations and a shaded area that depicts the standard deviation.

**Fig 5:**
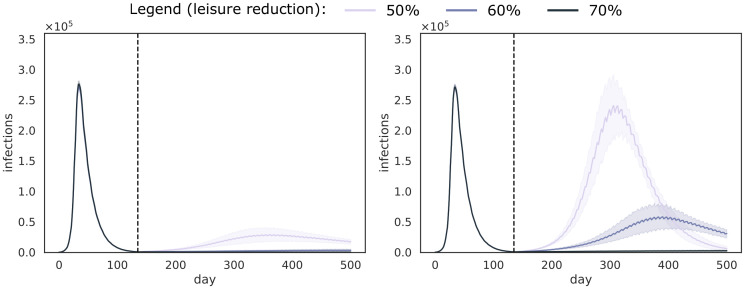
Trends for different leisure contact reductions, when performing weekly universal testing, and importing 10 cases per day. We assume that universal testing starts (and lock-down ends) on the first of July, as marked by the vertical dotted line, and that FNR_PCR_ = 0.1. We consider both isolation strategies: pool isolation (left panel) and individual isolation (right panel). The curves show a line that depicts the average over the trajectories of the result aggregations and a shaded area that depicts the standard deviation.

**Fig 6:**
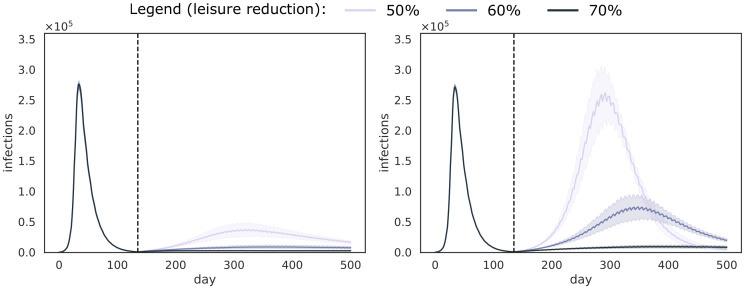
Trends for different leisure contact reductions, when performing weekly universal testing, and importing 50 cases per day. We assume that universal testing starts (and lock-down ends) on the first of July, as marked by the vertical dotted line, and that FNR_PCR_ = 0.1. We consider both isolation strategies: pool isolation (left panel) and individual isolation (right panel). The curves show a line that depicts the average over the trajectories of the result aggregations and a shaded area that depicts the standard deviation.

In the previous experiments, we consider a fixed leisure (post lock-down) contact reduction of 70%. To assess the amount of contacts that can be allowed when performing weekly universal testing, we investigate different leisure contact reductions, while keeping the work contact reduction fixed to 50%. To this end, in Fig 4, we show the simulation results for a leisure contact reduction of 50%, 60% and 70%, for both isolation strategies. To extend this setting to allow travel, we consider importing *n* cases per day, i.e., *n* individuals that were infected during a travel and return home. We show these results for respectively *n* = 10 and *n* = 50 in Figs 5 and 6. In Figs 4, 5 and 6 we show results for a false negative rate of 10%, and similar trends were observed for the 5% false negative rate (S1 Supplementary information S1.2). These results show, in simulation, that control of the epidemic can be achieved for leisure contact reductions up to 60%, when pool isolation is used. With individual isolation, the performance is lower, due to the additional test that needs to be performed, and the false negative rate that is associated with it. The pool isolation strategy can keep the epidemic at a reasonable level for a contact reduction of 50%, yet given this incidence level observed, this isolation strategy would require the isolation of many uninfected individuals, potentially impacting community compliance.

Note that we choose the same false negative rate for the pool test and the individual test, which is a conservative assumption, given that the individual test can be performed using the RNA extract that was established during the pool test. As the false negative rate compounds both errors with respect to sample collection and the sensitivity of the PCR test [42], it is reasonable to assume that the follow-up PCR test will have a lower false negative rate, as the virus was already extracted successfully from the individuals that are part of a positive pool. It is however challenging to choose a realistic reduction of the false negative rate for the additional test, as this is complicated when a pool is made up out of multiple positive samples and each of these samples can independently result in an error. Our experiments in S1 Supplementary information S1 show that a lower false negative rate results in improved performance when individual isolation is applied. Therefore, we believe follow-up research is warranted to obtain an improved estimate of the false negative rate of individual tests that use the RNA samples obtained from a pooled PCR test.

Our results also indicate that the importation of cases has an impact, which is more pronounced for lower leisure contact reductions, and when individual isolation is used. Overall, when the contact reduction is 70%, both isolation strategies are capable to control the epidemic.

In these experiments, we assume that when isolation is imposed, individuals are able to isolate from household members as well. When individuals are aware of their infection status, as is the case when individual isolation is applied, this assumption is reasonable and in line with earlier work [21]. However, we argue that this is less straightforward to accomplish in the case of pool isolation. Therefore, we challenge this assumption in S1 Supplementary information S1.3. These experiments show that the model trends are robust to this effect when the contact reduction is 70%. However this effect becomes pronounced when the number of contacts is increased (i.e., 50% and 60%). This highlights the importance of social distancing in the household, when a household member has been found positive.

To assess the impact of the PCR test reporting delay *d*_*t*_, we conduct a sensitivity analysis considering *d*_*t*_ = {1, 2, 3, 4} in S1 Supplementary information S1.4, and produce results in agreement with the work of Larremore et al. [43]. On the one hand, this sensitivity analysis shows that the pool isolation strategy is quite robust with respect to waiting times up to four days. On the other hand, our experiments show that for the individual isolation, the effect of this delay is much more pronounced and even dramatic for *d*_*t*_ ≥ 3. The reason for this strong effect is that, while individuals remain unaware of their infection status, as they are waiting for the test results to come in, it is possible that they infect their household members. Thus, while the original infected individuals can be identified and isolated, the newly infected individuals, which were not detectable yet, can cause new infections outside of the household. This effect is reduced when pool isolation is used, as the household members are part of the same pool and will thus be isolated as well. Based on this observation, we hypothesise that the use of *household isolation* (i.e., individual testing is used to determine the infection status of individuals and all households that contain infected individuals are isolated as a whole) could reduce this effect, which is confirmed experimentally in S1 Supplementary information S1.4.

## 4 Universal testing and “the hammer”

**Fig 7:**
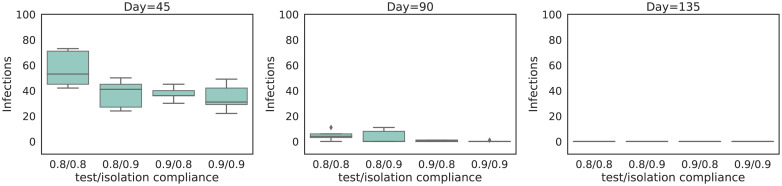
Distribution of the number of infections for the experiments when the lock-down restrictions are continued. We show different scenarios of compliance for testing and isolation. We show the number of infections at three different time points. i.e., 45 days (left panel), 90 days (middle panel) and 135 days (right panel) after the start of the universal testing procedure. These results consider a weekly universal testing procedure (i.e., *k* = 32 and *T*_*d*_ = 50000) and a FNR_PCR_ = 0.1, where the isolation strategy is individual isolation. Each box represents a combination of test and isolation compliance. Note that 135 days after the start of the universal testing procedure (not shown in this figure), the number of cases dropped to zero, for all the compliance scenarios.

In Section 3, the cases converge to zero over a time span that covers several months, given the leisure and work contact reductions that we assume. Here, we investigate the effect of weekly universal testing when it is conducted under contact reductions that are the same as during the lock-down that took place in Belgium during the first wave of the epidemic, i.e., a 75% workplace contact reduction and a 90% leisure contact reduction, also referred to as “the hammer”. In Fig 7, our simulation results show that the cases drop to zero quickly, even when considering lower levels of isolation and testing compliance.

While resistance towards lock-down measures has increased in many countries throughout the world during the COVID-19 pandemic, our intention is not to promote them here. Yet, this investigation does show that a (fast) local eradication might be feasible when performing a weekly universal testing procedure. Furthermore, this highlights the potential of universal testing strategies as a mitigation mechanism to other (future) emerging infectious diseases, that warrants further investigation.

## 5 Logistics of weekly universal testing

The time it takes to test the entire population (i.e., the sweep time), is directly proportional to the frequency at which each individual should be tested. Our experiments show that a sweep time of 1 week, of a population of 11 million individuals (i.e., using a fixed pool size *k* = 32 and a constant amount of tests *T*_*d*_ = 50k), yields the most promising results. We acknowledge that testing each individual on a weekly basis, requires a significant effort from the community. Yet, our simulation results do show that these actions may result in the control of the epidemic, even when much of the contact reductions are relieved, which might convince individuals that these measures are worth the effort. Notwithstanding, in order to make such a policy successful in a public health context, it is important that the societal awareness and support of these policies is stimulated, via prompt governmental communication.

Next to the efforts required by the population to get tested on a weekly basis, there is also a significant logistic challenge to obtain samples and ensure they can be tested in a reasonable amount of time. We discern four main considerations with respect to the logistics related to universal testing. Firstly, to enable a suitable geographical planning of the sample extraction and PCR testing, we argue that a decentralized approach will be most suitable. In our experiments, we already establish the test planning on a provincial level. In these provinces, a further division of this allocation towards regions that have PCR testing capabilities is possible, thereby facilitating a decentralized approach. As such, we argue that following a decentralized approach will enable to scale-up the testing infrastructure, without jeopardizing fast turn-around times of the PCR tests. Secondly, in order to perform sample extractions, a significant number of nursing staff members is required, for which we compute an estimate. For clarity, we express this computation in terms of a 1000 individuals, which can be extrapolated to the whole population in a straightforward way. When we assume that tests are collected over the time of 8 hours per day and every day of the week (including weekends), we need to test 18 individuals per hour, to test a set of 1000 individuals in one week. When we consider the use of a drive-through sample extraction facility, it is reasonable to assume that 1 nurse can test 9 individuals per hour, especially as a significant fraction of these individuals are part of the same household. When we assume that nurses work for 5 days per week and 8 hours per day, we conclude that we need on average 2.8 nurses per thousand individuals, to support a rapid turn-around time of the PCR tests. Thirdly, when the samples are extracted, they have to be pooled and PCR-tested using the available PCR-testing infrastructure. In our experiments, we assume that 50k PCR tests are available per day to test the sample pools. This assumption is in line with the capacity of the Belgian government to perform 70k PCR tests per day [22, 23]. Furthermore, this leaves 20k PCR tests to identify the individuals that are responsible for the positivity of the pool. Fourthly, we stress that it is important that the pools are tested in a reasonable amount of time. While there have been complaints from different countries (during the recent surges of SARS-CoV-2 in Europe an the United states, during the fall and winter of 2020) that the turn-around time of PCR testing in the context of contact tracing was too slow, we believe that the use of universal testing could improve this. This is the case when tests can be planned, rather than performed on demand (in the case of contact tracing), such that the burden on the testing infrastructure is distributed more uniformly. Furthermore, while in the context of contact tracing, sample extraction is commonly conducted by physicians and the extracted samples need to be sent to the PCR testing laboratories, which results in a lot of time spent on transporting the samples. To contrast this, the use of drive-through testing facilities, where sample collection and processing occurs in the same location, reduces the overall turn-around time for testing. Therefore, we believe that careful planning will enable to report the testing results within one day.

We acknowledge that the current testing practice, where a swab is placed in the nose, is quite invasive. In this regard, alternative sampling techniques, such as saliva sampling [44], should be considered. As saliva testing is a less invasive testing procedure, it would likely increase testing compliance. Furthermore, the use of saliva sampling could allow individuals to self-collect their samples, which could reduce the complexity of the testing logistics.

## 6 Related work

We take note of several works that relate to our method.

The work by de Wolff et al. also investigate pooling as a faster and more resource-efficient alternative to individual testing and present a method to determine which samples in a pool are positive using as little tests as possible [45]. Although in our work, we mostly focus on the phase of the epidemic where the number of infections is low, this technique could still be used in conjunction with the method that we propose.

The work by Taipale et al. considers a more accessible and cheaper, yet less reliable test, to infer whether individuals are infected [46]. This approach does rely on different chemical reagents than the ones currently kept in storage by governments. Therefore, implementing this method would require a change in the testing procedures, while the method that we propose could in principle be used right away [46], using the already implemented testing procedure and may therefore be utilized without further logistic alterations.

Recently, a platform, Swab-Seq, to perform massively scaled SARS-CoV-2 testing was introduced by Bloom et al. [47]. This technique allows to test thousands of samples simultaneously, by pooling these samples together in a well. This new technique is interesting, yet again, it still needs to be implemented in lab environments, to be used widely. As a side-note, while this scalability is impressive, we believe that a household-pooled testing approach could enable a faster turn-around time. On the one hand, for Swab-Seq to work for universal testing, thousands of samples need to be collected before the well is filled, and as Bloom et al. report, processing a well takes between 12 and 24 hours, resulting in a long overall turn-around time. On the other hand, the sample pooling approach we propose allows the extraction of samples that belong to a certain pool (e.g., *k* ∈ {16, 32}), after which this pool can be send directly for PCR testing.

The idea of fast and cheap antigen tests was introduced by Mina et al. (https://www.rapidtests.org), that aim to test individuals on a daily basis. The prospect of the wide availability of such tests is promising, and we believe that our modelling framework could prove useful to investigate the optimal allocation of such tests.

## 7 Discussion

In this study, we propose a method that renders universal testing feasible, requiring an amount of PCR tests per day, that is currently available, or will be made available to accommodate the expected increase in respiratory infections in autumn [24]. We evaluate this method using the individual-based epidemic model *STRIDE*, and investigate how this universal testing method can be used to control a local SARS-CoV-2 epidemic. This evaluation highlights two important results. Firstly, in simulation, our method for universal testing is able to keep the epidemic under control, when each individual can be tested on a weekly basis, even when many of the social contact restrictions are lifted (i.e., opening of schools, work contacts, leisure contacts). Secondly, in simulation, we show that, with stringent social contact reductions and without travel, the universal testing approach, allows for the number of infected individuals to converge to zero, rendering it a possible approach to eradicate SARS-CoV-2.

In this study, we use the *STRIDE* individual-based model, a mechanistic model that was fitted to data that was recorded during the Belgian SARS-CoV-2 epidemic [21]. The extensive validation in [21] shows that *STRIDE* allows to accurately model the transmission of SARS-CoV-2 within and among households. Our extension to the *STRIDE* model, with respect to universal testing, aims to reflect a realistic framework that can model the effect of difficulties and issues in the implementation of the intervention strategy, such as the false negative rate of the PCR test and difference in compliance, both with respect to isolation and testing. Nevertheless, we acknowledge that our epidemiological model is an abstraction of the real world that relies on a series of assumptions, and therefore the results presented in this manuscript should be interpreted with caution.

An important assumption in this work is the false negative rate of the PCR test, when a pool of samples is processed. We consider a range of false negative values (i.e., 5% and 10%), predominantly informed by the recent work of Yelin et al. [20]. This study associates the false negative rate to the pool size (i.e., more samples in the pool leads to a higher false negative rate), and attributes these differences to the effect of the dilution of the sample. While the work by Yelin et al. is valuable to inform our models [20], we argue that in order to implement pool-based testing on a large scale, a population-based evaluation of the sensitivity of pooled PCR testing is warranted.

To assess policies in a realistic framework, it is essential to consider difficulties and issues in the implementation of the intervention strategy, as they will be inevitable if the policy is to be implemented. This includes technical properties of the tests, as mentioned above, but also the willingness of individuals to participate. To this end, we validated our policies considering different levels of compliance with respect to testing and isolation. Unsurprisingly, our simulation results show that higher compliance results in a faster and more pronounced drop in the epidemic curve, yet overall our proposed strategy is robust to imperfect compliance. As stated earlier (see Section 5), we stress the importance to gain support from the community, to keep the population motivated to take part in this policy, especially if frequent testing is necessary. Next to compliance with respect to the testing policy, our simulations show that it is important for individuals to comply with the isolation policy in place. As a consequence, we note that lower assumptions on isolation compliance would have a substantial impact on the performance of universal testing procedures. In this study, we consider two isolation policies: pool isolation and individual isolation. While pool isolation reduces the need for additional tests, we acknowledge that it might be challenging for individuals to adhere to isolation when they are unaware of their infection status. From another perspective, as we aim to move to a low number of infections with our method, the number of pools that need to be isolated will also be low, and the burden of pool isolation might be acceptable. From a logistic perspective, individual isolation seems feasible when the incidence is low and the number of tests necessary to determine which individuals are positive is limited. For the Belgian case, 70k PCR tests were available by autumn of 2020 [23]. This would allow to test the population on a weekly basis (i.e., using 50k PCR tests and a pool size of 32), and have 20k PCR tests available to detect the infected individuals in the positive pools. We argue that individual isolation of cases would be beneficial with respect to isolation compliance. Analogously, the use of pool isolation could prove beneficial when the incidence is high (see top left panels of Fig 2 in Section 3), and the number of individuals of positive pools exceeds the number of available tests. This intervention strategy could be used to avert or shorten future lock-downs, where the isolation of pooled individuals is less burdensome compared to shutting down society in its entirety. Another option to reduce the number of individuals that need to be isolated when using pool isolation, is by adding an additional testing step to determine which household is positive. In contrast to individual isolation, this can be done by combing all samples that belong to the same household and test this pool of household members using a single PCR test. By pooling households members, we can determine which of the household(s) cause the positive test, and isolate this/these household(s) for 7 days. This way, the excessive number of additional tests is reduced to the number of households in the pool, which is a quantity we aim to minimize with our allocation algorithm (Section 2.1).

Next, we discuss the most important practical differences between contact tracing and universal testing. To ensure that contact tracing works, individuals need to be identified as an index case, index cases need to cooperate and share (and remember) their list of contacts, and all this needs to be achieved in as little time as possible [21]. Therefore, it might be challenging to fully control SARS-CoV-2 epidemics through the use of contact tracing [13–15]. In contrast, universal testing is logistically challenging, but once these logistics are in place, the process is transparent. An important consideration with respect to universal testing is compliance fatigue and this is an aspect that should be closely monitored. We note that this can be monitored, as the failure rate with respect to testing compliance is a by-product of the universal testing procedure.

In this work, we do not account for non-household populations explicitly (e.g., undocumented persons, homeless people, or visitors), and we acknowledge that it would be challenging to enrol non-household populations to the universal testing procedure. We argue that, from a modelling point of view, it is reasonable to assume that such populations are part of the non-compliant proportion of society. Furthermore, non-household individuals typically form only a small proportion of the total population [48] and therefore their impact on the general population will be limited. However, given the fact that this population is highly compartmentalized [49], and constitutes a fragile segment of the population, a specialized program is warranted to avoid outbreaks in this sub-population.

While we demonstrate the potential of universal testing to control local epidemics, there is another advantage, given that the procedure implicitly performs surveillance of the whole population. This means that a good estimate of the actual incidence, and the geographic distribution of this incidence, will be available at any time. This way, emergency signals will be picked up more rapidly, enabling a swift response that might avoid more invasive control measures.

To achieve durable control, our modelling experiments show that sustained universal testing programs are necessary, rather than a single testing sweep, that was recently used in Slovakia [50, 51]. As a result, the economic cost of sustained universal testing will be an important determinant. We note that pool-based testing can significantly reduce the costs associated with mass testing, and therefore, the economic costs will be mostly related to recruit nurses, infrastructure to extract samples and infrastructure to communicate test results. Adjacent to this, we acknowledge that the number of individuals that need to be isolated is important, both with respect to compliance and from an economic perspective. In this regard, we believe it is important to have two distinct isolation strategies available, i.e., pool isolation versus individual isolation. On the one hand, the pool isolation strategy has the economic disadvantage that many (potentially uninfected) individuals need to be isolated. However, when incidence is high, it could enable the release of more stringent measures, thereby rendering it economically interesting again. On the other hand, the individual isolation strategy allows for a more directed isolation approach, thereby rendering it economically more interesting, when incidence level allows for the strategy to be used. In general, we acknowledge that a detailed health economic assessment is warranted to carefully assess the economic and societal potential of the universal testing procedure.

We also show, in simulation, that our method has the potential to eradicate SARS-CoV-2 in a local setting. While in our simulations, we investigate the effect of importing cases (i.e., infected individuals that return from a travel), we limit ourselves to a constant number of daily importation events. The implementation in a larger travel union (e.g. European Economic Area, United states of America) would be more challenging, as different countries have different epidemic states, and the travel flows between these countries should be considered. Yet, when the countries in a travel union coordinate and implement the system of universal testing locally, the eradication of SARS-CoV-2 within the travel union could be conceivable, when importations from outside the travel union are carefully monitored (e.g., testing on the airports).

**Fig 8:**
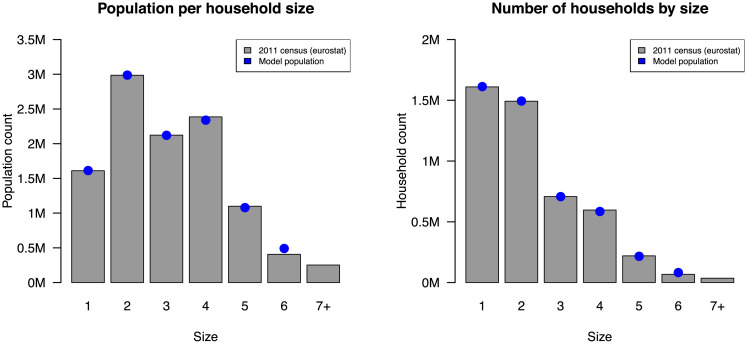
Population size per household size and number of households per size: Belgian 2011 census and model population. Numbers are expressed in million (M). Figure from Willem et al. [21].

The household-to-pool allocation algorithm that we introduce in Section 2.1 is guided by a simple heuristic that results in an optimal allocation, i.e., to adequately fill up the pools and to minimize the difference in number of households between pools (Section 2.1, Eq 4). However, there are some border cases, where using the heuristic could result in an allocation that exceeds the capacity of some of the pools. We argue that such border cases are unlikely to occur in a population that is large enough (i.e., it thus involves a large number of bins) and has a realistic distribution of household sizes, and for pools that have sufficient capacity. The intuition behind this is that, as we add households ordered by their size, first the largest households that are most likely to generate an overflow are assigned. Therefore, as long as there is a sufficient number of smaller households, which is expected and observed in our household dataset (see Fig 8), these households will act as padding to fill up the pools. We were unable to come up with realistic counter-examples, and in our experiments, no overflows were detected.

One point of concern is that recently recovered individuals could still carry virus particles and render a PCR test positive. While this is not a problem when individual isolation is used, as these individuals can be excluded from the future pool, this could result in false positives when pool isolation is used. While we argue that this artefact is less likely to occur due to sample dilution [20], this warrants further investigation. We note that, if problematic, this issue could be mitigated by pooling households to determine which of the households was infected, as we discussed earlier.

In this work, we present a modelling framework to investigate the impact of universal testing. We use this framework to perform a qualitative investigation of the feasibility of universal testing. Currently, we assume uniform distributions for our model parameters, as specified in Table 2. While these distributions are a conservative assumption, as the uniform distribution maximizes entropy over a specified interval, we note that the use of a log-normal or gamma distribution for the incubation period would be interesting to investigate for future work [31, 34]. Uniform distributions induce an increase in transmissibility during the pre-symptomatic phase, possibly reducing the effectiveness of the proposed strategy, as by the time a case is detected it might have already generated a new infection. While this will not have an important effect on the qualitative results of the study, it might alter the quantitative results.

We show in our simulation experiments that the impact of leisure contact reduction has a significant impact on the effectiveness of the weekly universal testing approach. To this end, we conduct a sensitivity analysis considering different levels of leisure contact reduction, yet it remains to be decided how such contact reductions can be translated to a public health context. In this regard, the use of social bubbles of different constellations could be investigated [21], but also the effect of additional hygienic measures (e.g., mouth masks) to reduce the risk of certain contacts could be assessed [52, 53].

Finally, we note that this study was conducted in the context of the Belgian SARS-CoV-2 epidemic, and the reader should be careful to extrapolate these findings to other epidemic contexts. Nevertheless, when the population structure (e.g., household distribution) and size are similar, we believe that the methods presented in this work can be applied to administrative regions of different countries. Furthermore, in this study, we assume 70k PCR tests per day for a population of 11 million (50k PCR tests for weekly testing, and 20k PCR tests to test the individuals in the positive pools), which implies that every day a number of tests is available that corresponds to 0.68% of the population. This number of tests is a reasonable assumption, to which many countries anticipated for the autumn and winter of 2020 [22, 24]. While the logistic requirements to establish weekly universal testing are challenging, we argue and motivate (Section 5) that a decentralized approach is feasible. Furthermore, testing methods such as saliva testing could further reduce the threshold to facilitate universal testing. Acknowledging these complications, we demonstrate that the benefits of universal testing are significant and can result in an improved control and surveillance of the epidemic, resulting in an increase in leisure contacts and an overall societal and economic relaxation.

## 8 Conclusion

In this work, we present a new method to approach universal testing, where we combine households to form sample pools, such that with the available testing capacity, it is possible to test the whole Belgian population on a weekly basis. This weekly universal testing approach allows to isolate pools (when the incidence is high) or to identify and isolate the individuals that are actually positive, using a follow up test (when the incidence is low). On the one hand, such a universal testing approach presents several logistic challenges, which we discuss and for which we formulate a logistic framework. On the other hand, we show in an individual-based model that this mitigation strategy allows an increase in the number of contacts (e.g., work, leisure, schools) and is robust with respect to the importation of cases via travel. We conclude that weekly universal testing could prove an additional strategy to control SARS-CoV-2 outbreaks. Furthermore, we show that the use of universal testing in combination with stringent contact reductions could be considered as a strategy to eradicate the virus. To allow for weekly universal testing to be used in practice, our robustness analyses show that both compliance support from the community and an adequate organisation of sampling logistics are crucial.

## Supporting information

S1 Supplementary information This Supporting Information document (S1) contains additional modelling results not shown in the main manuscript and covers a series of sensitivity analyses to support our experiments. (PDF)
