# Supplementary material for: Assessing the feasibility and effectiveness of household-pooled universal testing to control COVID-19 epidemics
Source: PLoS Comput Biol. 2021 Mar 9;17(3):e1008688. doi: 10.1371/journal.pcbi.1008688 (PMC7943003; doi:10.1371/journal.pcbi.1008688)
Supplement: S1 Supplementary information — (PDF) [file pcbi.1008688.s001.pdf]

Assessing the feasibility and effectiveness of household-pooled universal testing to control COVID-19 epidemics: Supplementary Information

Pieter J.K. Libin[1,2,3], Lander Willem[4], Timothy Verstraeten[2], Joris Vanderlocht[1], Andrea Torneri[1], and Niel Hens[1,4]

[1]Interuniversity Institute of Biostatistics and statistical Bioinformatics, Data Science Institute, Hasselt University, Hasselt, Belgium
[2]Artificial Intelligence Lab, Department of computer science, Vrije Universiteit Brussel, Brussels, Belgium
[3]KU Leuven – University of Leuven, Department of Microbiology and Immunology, Rega Institute for Medical Research, Clinical and Epidemiological Virology, Leuven, Belgium
[4]Centre for Health Economics Research and Modelling Infectious Diseases, Vaccine and Infectious Disease Institute, University of Antwerp, Antwerp, Belgium

1 Model results for $\mathbf{FNR_{PCR}} = 0.05$

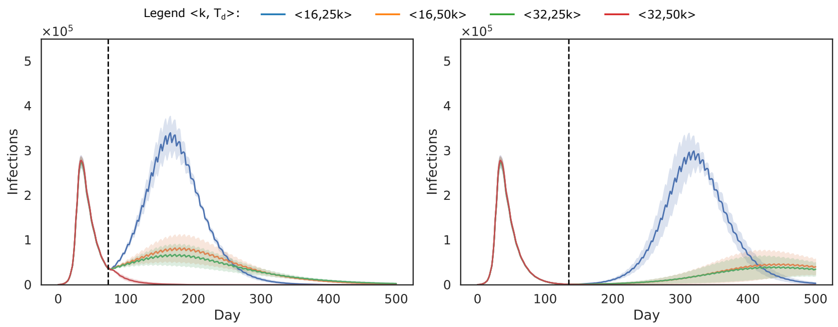

Figure 1: **Trends for pool isolation.** Simulated epidemic curves for all combinations of parameters $\langle k, T_d \rangle$, for $\text{FNR}_{\text{PCR}} = 0.05$. Universal testing starts at the first of May (left panel) and the first of July (right panel), as indicated by the vertical dotted line. This vertical line also marks the start of the universal testing procedure. We follow the isolation strategy where we isolate all individuals that are part of an infected pool. The curves show a line that depicts the average over the trajectories of the result aggregations and a shaded area that depicts the standard deviation.

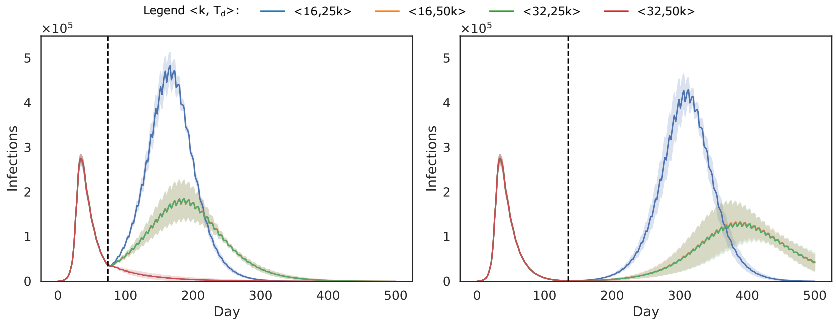

Figure 2: **Trends for individual isolation.** Simulated epidemic curves for all combinations of parameters $\langle k, T_d \rangle$, for $\text{FNR}_{\text{PCR}} = 0.05$. Universal testing starts at the first of May (left panel) and the first of July (right panel), as indicated by the vertical dotted line. This vertical line also marks the start of the universal testing procedure. We follow the isolation strategy where we identify the infected individuals in the positive pool. The curves show a line that depicts the average over the trajectories of the result aggregations and a shaded area that depicts the standard deviation.

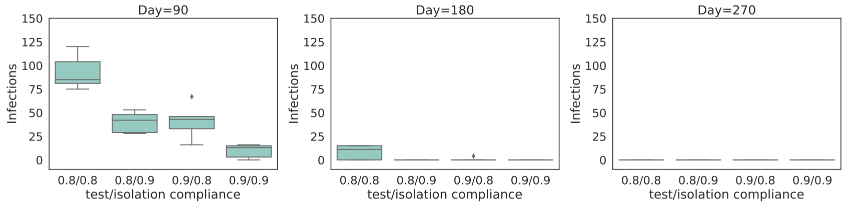

Figure 3: **Distribution for pool isolation.** Distribution of the number of infections for the experiment when the lock-down end on the first of July, in different scenarios of compliance for testing and isolation. We show the number of infections at three different time points. i.e., 90 days (left panel), 180 days (middle panel) and 270 days (right panel) after the start of the universal testing procedure. These results consider a weekly universal testing procedure (i.e., $k = 32$ and $T_d = 50000$) and a $\text{FNR}_{\text{PCR}} = 0.05$, where the isolation strategy is pool isolation. Each box represents a combination of test and isolation compliance.

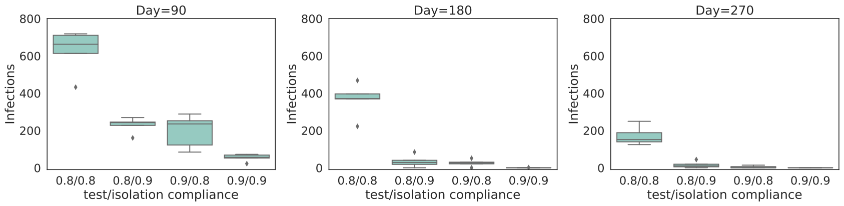

Figure 4: **Distribution for individual isolation.** Distribution of the number of infections for the experiment when the lock-down end on the first of July, in different scenarios of compliance for testing and isolation. We show the number of infections at three different time points. i.e., 90 days (left panel), 180 days (middle panel) and 270 days (right panel) after the start of the universal testing procedure. These results consider a weekly universal testing procedure (i.e., $k = 32$ and $T_d = 50000$) and a $\text{FNR}_{\text{PCR}} = 0.05$, where the isolation strategy is individual isolation. Each box represents a combination of test and isolation compliance.

2 Model results for different leisure reductions $\mathrm{FNR}_{\mathrm{PCR}} = 0.05$

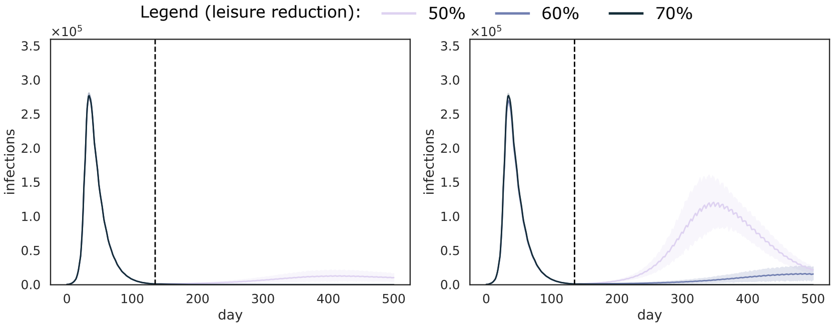

Figure 5: **Simulated epidemic curves for different leisure contact reductions, when performing weekly universal testing.** We assume that universal testing starts on the first of July and that $\mathrm{FNR}_{\mathrm{PCR}} = 0.05$, as indicated by the vertical dotted line. This vertical line also marks the start of the universal testing procedure. We consider both isolation strategies: pool isolation (left panel) and individual isolation (right panel). The curves show a line that depicts the average over the trajectories of the result aggregations and a shaded area that depicts the standard deviation.

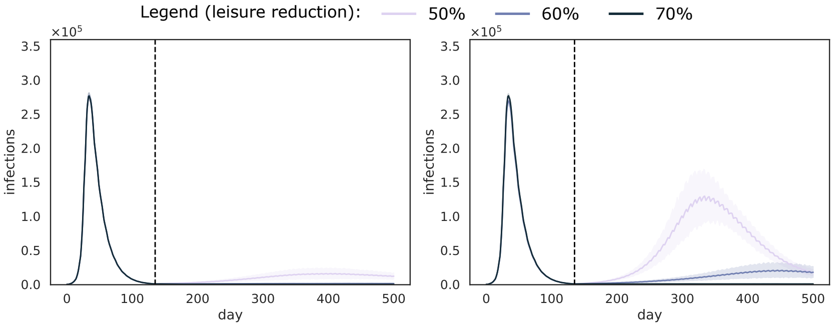

Figure 6: **Simulated epidemic curves for different leisure contact reductions, when performing weekly universal testing, and importing 10 cases per day.** We assume that universal testing starts on the first of July and that $\mathrm{FNR}_{\mathrm{PCR}} = 0.05$, as indicated by the vertical dotted line. This vertical line also marks the start of the universal testing procedure. We consider both isolation strategies: pool isolation (left panel) and individual isolation (right panel). The curves show a line that depicts the average over the trajectories of the result aggregations and a shaded area that depicts the standard deviation.

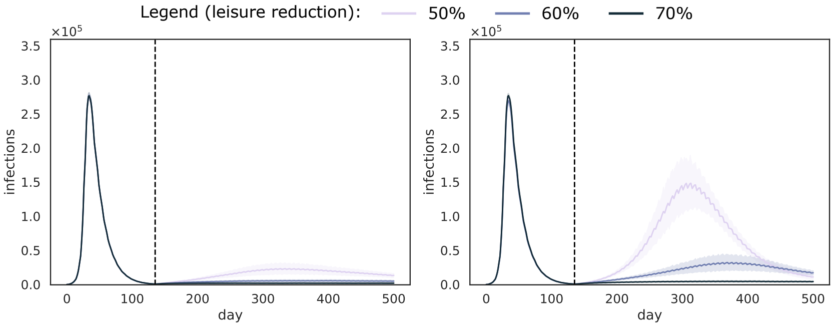

Figure 7: **Simulated epidemic curves for different leisure contact reductions, when performing weekly universal testing, and importing 50 cases per day.** We assume that universal testing starts on the first of July and that $\mathrm{FNR}_{\mathrm{PCR}} = 0.05$, as indicated by the vertical dotted line. This vertical line also marks the start of the universal testing procedure. We consider both isolation strategies: pool isolation (left panel) and individual isolation (right panel). The curves show a line that depicts the average over the trajectories of the result aggregations and a shaded area that depicts the standard deviation.

3 Model results for the household isolation challenge

In the main experiments, we assume that when isolation is imposed, individuals are able to isolate from household members as well. When individuals are aware of their infection status, as is the case when individual isolation is applied, this assumption is reasonable and in line with earlier work [1]. However, we argue that this is less straightforward to accomplish in the case of pool isolation. Therefore, in this appendix, we challenge this assumption.

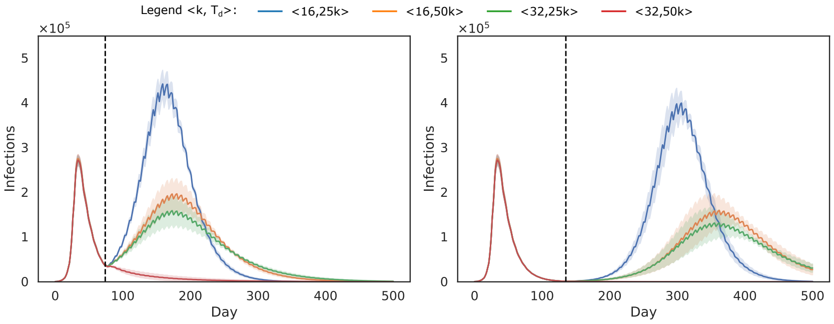

Figure 8: Trends for all combinations of parameters $\langle k, T_d \rangle$, for $\text{FNR}_{\text{PCR}} = 0.1$. Universal testing starts at the first of May (left panel) and the first of July (right panel). We follow the pool isolation strategy, where we isolate all individuals that are part of an infected pool. The curves show a line that depicts the average over the trajectories of the result aggregations and a shaded area that depicts the standard deviation.

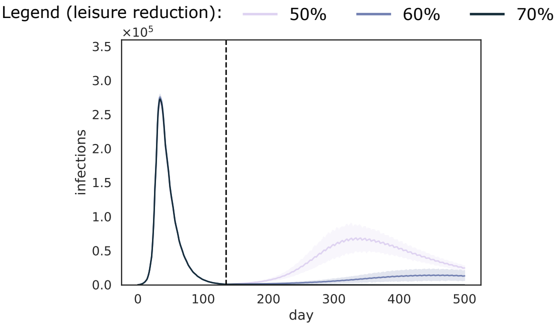

Figure 9: Trends for different leisure contact reductions, when performing weekly universal testing. We assume that universal testing starts on the first of July and that $\text{FNR}_{\text{PCR}} = 0.1$. We consider pool isolation and challenge the assumption of household isolation. The curves show a line that depicts the average over the trajectories of the result aggregations and a shaded area that depicts the standard deviation.

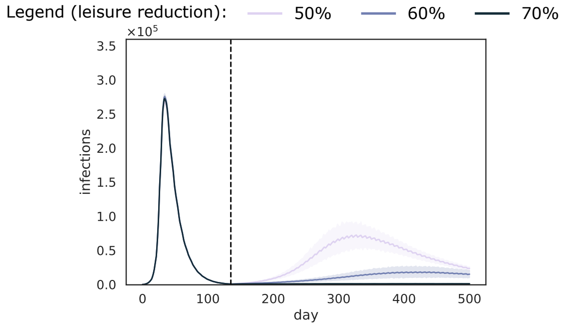

Figure 10: Trends for different leisure contact reductions, when performing weekly universal testing, and importing 10 cases per day. We assume that universal testing starts on the first of July and that $\text{FNR}_{\text{PCR}} = 0.1$. We consider pool isolation and challenge the assumption of household isolation. The curves show a line that depicts the average over the trajectories of the result aggregations and a shaded area that depicts the standard deviation.

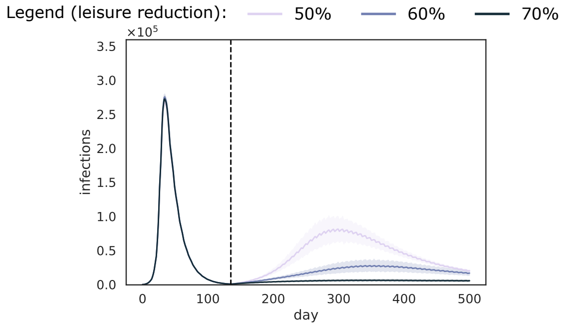

Figure 11: Trends for different leisure contact reductions, when performing weekly universal testing, and importing 50 cases per day. We assume that universal testing starts on the first of July and that $\text{FNR}_{\text{PCR}} = 0.1$. We consider pool isolation and challenge the assumption of household isolation. The curves show a line that depicts the average over the trajectories of the result aggregations and a shaded area that depicts the standard deviation.

4 PCR test reporting delay (d_t) sensitivity analysis

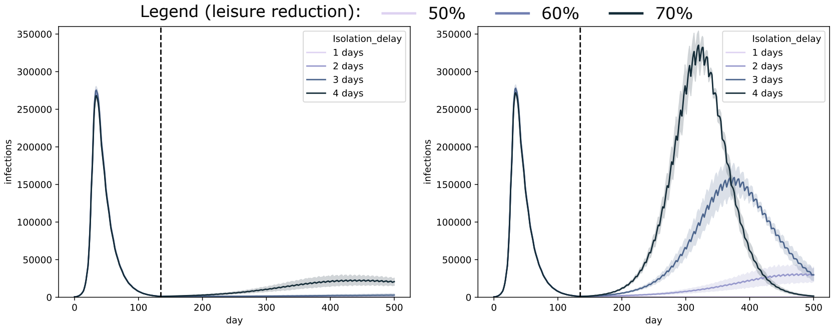

Figure 12: Sensitivity analysis for weekly universal testing ($k = 32$, $T_d = 50$k) with a contact reduction of 70%, considering $d_t = \{1, 2, 3, 4\}$. We assume that universal testing starts on the first of July and that $\text{FNR}_{\text{PCR}} = 0.1$. We consider both isolation strategies: pool isolation (left panel) and individual isolation (right panel). The curves show a line that depicts the average over the trajectories of the result aggregations and a shaded area that depicts the standard deviation.

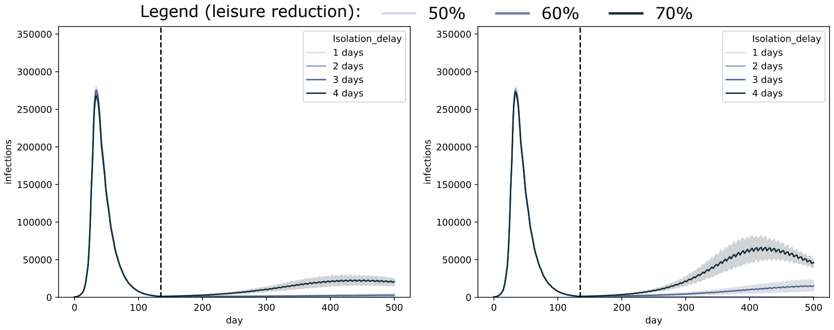

Figure 13: Sensitivity analysis for weekly universal testing ($k = 32$, $T_d = 50$k) with a contact reduction of 70%, considering $d_t = \{1, 2, 3, 4\}$. We assume that universal testing starts on the first of July and that $\text{FNR}_{\text{PCR}} = 0.1$. We consider both isolation strategies: pool isolation (left panel) and household isolation (right panel). The curves show a line that depicts the average over the trajectories of the result aggregations and a shaded area that depicts the standard deviation.

5 Weekly versus bi-weekly universal testing

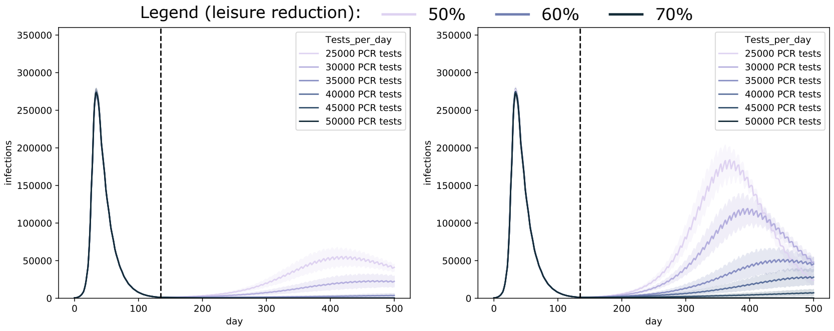

Figure 14: Weekly versus bi-weekly universal testing (i.e., $k = 32, T_d \in \{25k, 30\text{k}, 35\text{k}, 40\text{k}, 45\text{k}, 50\text{k}\}$) with a contact reduction of 70%, considering $d_t = \{1, 2, 3, 4\}$. We assume that universal testing starts on the first of July and that $\text{FNR}_{\text{PCR}} = 0.1$. We consider both isolation strategies: pool isolation (left panel) and individual isolation (right panel). The curves show a line that depicts the average over the trajectories of the result aggregations and a shaded area that depicts the standard deviation.

References

[1] L. Willem, S. Abrams, P. Libin, P. Coletti, E. Kuylen, O. Petrof, S. Mogelmose, J. Wambua, S. A. Herzog, C. Faes, et al. The impact of contact tracing and household bubbles on deconfinement strategies for COVID-19: an individual-based modelling study. *Nature Communications*, 2020, In press.
